# Follicular-fluid extracellular vesicles support energy metabolism of bovine oocytes, improving blastocyst development and quality^†^

**DOI:** 10.1093/biolre/ioaf096

**Published:** 2025-04-24

**Authors:** Paulina Lipinska, Katrien Smits, Ann Van Soom, Krishna Chaitanya Pavani, Ewelina Warzych

**Affiliations:** Department of Genetics and Animal Breeding, Poznan University of Life Sciences, Poznan, Poland; Department of Internal Medicine, Reproduction and Population Medicine, Ghent University, Merelbeke, Belgium; Department of Internal Medicine, Reproduction and Population Medicine, Ghent University, Merelbeke, Belgium; Department of Internal Medicine, Reproduction and Population Medicine, Ghent University, Merelbeke, Belgium; Department for Reproductive Medicine, Ghent University Hospital, Gent, Belgium; Department of Genetics and Animal Breeding, Poznan University of Life Sciences, Poznan, Poland

**Keywords:** extracellular vesicles, oocyte, embryo, energy metabolism

## Abstract

Extracellular vesicles (EVs) from follicular fluid (FF) seem to play a significant role in communication within ovarian follicles in several species. The present study aimed to examine the supporting effect of FF-derived small EVs (FF-sEVs) during in vitro maturation (IVM) of bovine cumulus–oocyte complexes (COCs) under conditions of disturbed energy metabolism. Bovine COCs were matured in vitro with inhibitors targeting lipid metabolism (etomoxir) or glucose metabolism (iodoacetate combined with dehydroepiandrosterone), in the presence or absence of FF-sEVs. Following maturation, oocytes and cumulus cells were analyzed by real-time quantitative polymerase chain reaction (qPCR) and stained to visualize lipid droplets. The uptake of FF-sEVs was visualized by fluorescent labeling. In vitro fertilization and embryo culture were followed by mass spectrometry analysis of hatched blastocysts. We demonstrate for the first time that FF-sEVs are transported from the medium into the oocytes, via the cumulus cells and through transzonal projections into the perivitelline space and ooplasm. Cumulus cells under metabolic stress conditions exhibit an increased FF-sEV uptake from the maturation medium. FF-sEV supplementation during metabolic stress conditions enhances the MII rate in oocytes and positively affects subsequent embryo development and quality revealed by altered metabolic activity. Lipid droplet parameters and gene expression in cumulus cells and oocytes are affected by FF-sEV supplementation, which is more pronounced in cumulus cells. Our findings show that FF-sEV supplementation during IVM under metabolic stress conditions significantly affects COCs, with a positive effect on further blastocyst quality. We provide novel insights into the role of FF-sEVs in oocyte maturation and blastocyst development.

## Teaser

Follicular fluid sEV supplementation during IVM under metabolic stress conditions significantly affects COCs, with a positive effect on blastocyst quality, which provides novel insights into the role of EVs in oocyte and embryo development.

## Graphical Abstract

**Graphical Abstract:**
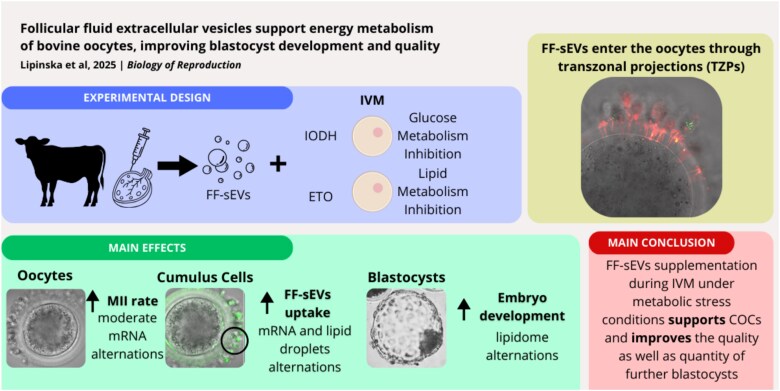

## Introduction

Over the past decades, significant progress in assisted reproductive technologies has been made not only to treat human infertility but also to support animal breeding and preserve genetic material from valuable animals and endangered species. However, in vitro embryo production still falls short of replicating natural conditions since the final outcome is not fully satisfactory. With regard to farm animal species, the average blastocyst rate is 30%–40% for cattle, 20%–40% for horses, and 30%–50% for pigs [1]. The suboptimal results are partly due to deficiencies of oocytes in vitro maturation (IVM) systems, which do not fully mimic in vivo follicular conditions. Oocyte quality, reflected by its intrinsic developmental potential, determines its ability to be fertilized and develop into a healthy embryo and offspring. Poor oocyte quality may lead to arrested embryo development [2], partially due to disturbances of key metabolic pathways. Well-balanced oocyte energy metabolism has a significant impact on oocytes as well as further embryo development [3].

Intercellular communication within the ovarian follicle is crucial for the development of the competent oocyte [4]. A well-organized follicular environment is based on close cooperation among oocyte, cumulus cells (CCs), and follicular fluid (FF) [5]. Follicular fluid is the natural medium where the oocyte grows and matures. Numerous biomolecules, such as metabolites, growth factors, hormones, and extracellular vesicles (EVs), are abundant in FF [6, 7]. These components play a crucial role in supporting oocyte maturation by facilitating the bidirectional communication between oocytes and somatic cells during follicle development [8]. Among the various factors involved in this phenomenon, EVs derived from FF (FF-EVs) have gained increasing scientific attention. Extracellular vesicles are groups of lipid bilayer nanoparticles, ranging in size from 50 to 300 nm, that are secreted by different cell types [9, 10]. These particles transfer specific cargo to their target cells, where they impact paracrine signaling in healthy and pathological processes. Studies in humans, cattle, and horses demonstrated that FF-EVs affect the transcriptome and proteome of CCs and oocytes, as well as oocyte developmental potential [11–13]. It has been shown that FF-EV supplementation modulates CC expansion during IVM of bovine oocytes [14]. Moreover, the impact of FF-EVs on bovine oocytes maturing under unfavorable conditions such as heat shock, oxidative stress, or after vitrification-warming has been described, causing better oocyte resistance to applied stress factors [11, 15–18]. Overall, a beneficial impact of FF-EVs on the oocyte has been shown, but their specific role in oocyte metabolism remains to be determined.

There are two main metabolic sources of energy for cells—glucose and lipids. Oocytes demonstrate limited ability of glucose uptake, which results from the lack of expression of the high-affinity, insulin-regulated glucose transporter SLC2A4 (or GLUT4) [19]. Consequently, they depend on CCs, which absorb glucose from the environment, convert it into pyruvate and lactate, and pass these products on to the oocyte cytoplasm [20]. The metabolism of lipids, which is an alternative energy source, is carried out by β-oxidation in the mitochondria in both oocytes and CCs. Lipids can be supplied to the oocyte from internal storage within the cumulus–oocyte complex—COC (mainly from lipid droplets [LDs]) or from external energy sources—FF or IVM medium [21]. The cleavage-stage embryo uses pyruvate as a major energy source, whereas, after compaction, glucose consumption increases. However, embryos at different steps of development may also utilize fatty acids to produce energy [22]. It has been shown that disturbance of energy metabolic homeostasis causes significant changes in the oocyte competence and quality [3, 23]. The energy metabolism is controlled by specific genes, and several were selected for the present experiment. The fatty acid metabolism–related genes [Fatty Acid Synthase (*FASN),* Peroxisome Proliferator-Activated Receptor α *(PPARA),* Perilipin 2 *(PLIN2*)] are involved in lipid synthesis, storage, and oxidation, which are essential for cellular energy homeostasis [24–26]. The glucose metabolism–related genes [Acetyl-CoA Carboxylase α (*ACACA),* Glucose Transporter 1 *(GLUT1*)] play crucial roles in glucose uptake and lipid biosynthesis, both fundamental for energy balance and metabolic activity [27, 28]. Additionally, *GDF9* (Growth Differentiation Factor-9) is a marker of oocyte competence, reflecting the metabolic status and developmental potential of cells [29].

In the present study, we showed that FF-sEVs (small EVs of diameter < 200 nm) supplementation during IVM under metabolic stress conditions supports energy metabolism in COCs, improving further embryo development and quality. Moreover, for the first time, we revealed that FF-sEVs enter the oocyte via transzonal projections (TZPs) present in the zona pellucida.

## Materials and methods

The study was conducted according to the Polish Act of the protection of animals used for educational or scientific purposes of 15 January 2015 and directive 2010/63/EU (Journal of Laws Dz.U. 2015 No. item 266) of the European Parliament of 22 September 2010 (2010/63/EU) on the protection of animals used for scientific objectives, so this study did not require the consent of the competent ethics committee for animal experiments. The biological material (bovine ovaries) was obtained from commercial slaughterhouses after routine slaughter. Ovaries were transported from the slaughterhouse within 2 h in a thermal bag at ~30°C.

All relevant data of the experiments were submitted to the EV-TRACK knowledgebase (EV-TRACK ID: EV250005) [30].

Unless otherwise stated, all reagents were supplied by Merck Group.

### Collection of follicular fluid

Bovine FF collection was carried out according to Azari-Dolatabad et al. [31]. Briefly, FF was collected from ovarian follicles (4–8 mm in diameter) in six replicates. Ovaries were washed three times with warm saline solution, sterilized with 90% ethanol, and then dried with clean towels. Six batches of FF were collected from a mix of Belgian Blue and Holstein cow ovaries on six different, random days. The specific breed proportion, origin, age, and metabolic status of the cows were not recorded. Ovaries were selected based on the following criteria at the individual ovary level: (1) absence of corpora lutea, (2) absence of follicles greater than 25 mm in diameter, and (3) normal morphological characteristics, such as absence of adhesions and scars. Follicular fluid was aspirated from follicles with a sterile syringe and 1.2 mm needle. Acquired samples (1 mL per replicate) were then centrifuged at 4°C, 2000 g for 10 min. The supernatant was filtered with a 0.22 μm filter to remove cell debris from the fluid, which resulted in further isolation of small EVs (FF-sEVs) of diameter < 220 nm, and it was transferred into 2 mL cryovials and stored at −80°C [31].

### Extracellular vesicle isolation

Extracellular vesicle isolation from FF was carried out as described [4]. In brief, iodixanol working solution was prepared by adding a solution buffer [60 mM Tris-HCl, 6 mM EDTA, and 0.25 M sucrose (pH 7.4)] to a stock solution of OptiPrep Density Gradient Medium (Sigma-Aldrich). Then, the homogenization buffer [10 mM Tris-HCl, 1 mM EDTA, and 0.25 M sucrose (pH 7.4)] was combined with iodixanol working solution to create gradients with 5%, 10%, 20%, and 40%. The gradient was created in a 16.8 mL open-top polyallomer tube (Beckman Coulter, Brea, CA, USA) by layering 4 mL of 40%, 4 mL of 20%, 4 mL of 10%, and 3.5 mL of 5% solutions on top of each other. One-milliliter FF samples were layered on top of prepared gradients. Then, samples were centrifuged at 4°C for 18 h at 100 000 *g* (SW 32.1 Ti rotor, Beckman Coulter, Brea, CA, USA). Gradient fractions of 1 mL were collected starting from the top of the gradient. Fractions 9 and 10, corresponding to a density of 1.087–1.109 g/mL, were pooled, diluted in phosphate buffered saline (PBS), and centrifuged for 3 h at 100 000 *g* and 4°C. Obtained pellets of FF-sEVs from each replicate were pooled and resuspended in PBS to a final concentration of 98.7 mg/mL, as measured by NanoDrop, and stored at −80°C. Subsequent characterization of the EVs was performed by Nanoparticle Tracking Analysis (NTA), transmission electron microscopy (TEM), and Western blotting (WB).

### Nanoparticle tracking analysis

To assess the size and the concentration of small EVs isolated from FF, Nanoparticle Tracking Analysis (NTA) was conducted with a NanoSight LM10 microscope. Six batches of isolated FF-sEVs were mixed and diluted in PBS to achieve a particle concentration of about 1 × 10^8^–1 × 10^9^ particles/mL. Samples were briefly vortexed and injected into the measurement chamber. Three individual videos were recorded for the sample and analyzed by NTA (NTA 3.4—Sample Assistant Build 3.4.4—SA) with a cCMOS camera type, a Blue488 laser type, an FPS of 25.0, and 749 frames.

### Transmission electron microscopy

Small EVs derived from FF underwent morphological identification through TEM as detailed previously [4, 32]. Briefly, each sample was placed on electron microscopy grids precoated with formvar/carbon support film (FCF200H-CU-TB; Aurion). The grids were then stained with 1% uranyl acetate (in double-distilled water) for 45 s. Electron microscopy (JEM 1400 plus, JEOL, Benelux; and Zeiss EM 109, Carl Zeiss) was used to examine the prepared grids. Images were captured using a Quemasa charge-coupled device camera (Olympus Soft Imaging Solutions GMBH).

### Western blotting

In order to confirm the presence of FF-sEVs in the samples, WB was conducted using markers specific to EVs: CD63, CD9, and TSG101 (EV-associated proteins). All samples of isolated FF-sEVs were first dissolved in a reducing buffer [0.005% bromophenol blue, 3% 2-mercaptoethanol, 9.2% SDS, 40% glycerol, and 0.5-M Tris-HCl (pH 6.8)] and heated for 5 min at 95°C. SDS polyacrylamide gel electrophoresis was used to separate proteins. Samples were transferred into nitrocellulose membranes that were subsequently blocked with 5% BSA + 0.5% Tween for 45 min at room temperature. Next, the membrane was exposed to CD63 (1:200 in 5% BSA + 0.5% Tween PBS, ab68418, Abcam), CD9 rabbit (1:1000 in 5% BSA + 0.5% Tween PBS, CST- D3H4P, Cell Signaling Technology), and TSG101 mouse (1:1000 in 5% BSA + 0.5% Tween PBS, sc-7964) primary antibodies at 4°C and incubated overnight. Next, membranes were washed with 0.5% Tween in PBS followed by incubation with specific secondary antibodies all conjugated with horseradish peroxidase—HRP [anti-mouse IgG (GE Healthcare UK), 1:3000 in 5% milk +0.5% Tween PBS; anti-rabbit IgG (GE Healthcare UK), 1:4000 in 5% BSA + 0.5% Tween PBS]. After a final wash step, a chemiluminescence substrate (WesternBright Sirius, Advansta) was added to the membranes. Imaging was performed using Proxima 2850 Imager (IsoGen Life Sciences).

### Collection of bovine cumulus–oocyte complexes

Ovaries were collected in the slaughterhouse and transported to the laboratory. Good-quality COCs with no cytoplasmic degradation and at least three to four layers of CCs closely attached to the zona pellucida were acquired through aspiration using a 1.2 mm needle and syringe from ovarian follicles of 4–8 mm diameter.

### In vitro maturation

Cumulus-oocytes complexes (50–60 COCs per one drop) were matured in 500 μL of TCM199 + Glutamax (Gibco, Thermo Fisher Scientific, MA, United States) medium supplemented with 6 mg/mL fafBSA (fatty acid–free bovine serum albumin), 0.25 mM Na pyruvate, 1× concentrated penicillin–streptomycin solution, 2 μg/mL follicle stimulating hormone (FSH), and 1 μg/mL β-estradiol for 22 h. The conditions were maintained at 39°C, 5% CO_2_, and maximum humidity.

Besides the control group, two treatments were included to inhibit glucose or fatty acid metabolism. All three treatments were supplemented with FF-sEVs or not, resulting in a total of six experimental groups:

1) CON—un-supplemented control group with IVM in the basic medium;

2) IODH, where IVM medium was supplemented with two inhibitors of glucose metabolism [1.5 μM iodoacetate (IO, inhibitor of glycolysis) diluted in water and 150 μM dehydroepiandrosterone (DHEA, inhibitor of pentose phosphate pathway) diluted in DMSO (dimethyl sulfoxide)];

3) ETO, where IVM medium was supplemented with an inhibitor of fatty acid metabolism (150 μM etomoxir diluted in DMSO);

4) CON+, where IVM medium was supplemented with 6.5 μg/mL of FF-sEVs;

5) IODH+, where IVM medium was supplemented with IO and DHEA as well as 6.5 μg/mL of FF-sEVs; and

6) ETO+, where IVM medium was supplemented with etomoxir as well as 6.5 μg/mL of FF-sEVs.

The concentration of DMSO in the IVM medium was 0.1%. This concentration was previously shown not to affect further embryo development [33]. Follicular-fluid sEVs were supplemented in a volume of 10 μL per IVM well (stock concentration—98.7 mg/mL). The concentration was calculated according to the previously published guidelines [34].

After maturation, COCs were either denuded and fixed for further procedures or fertilized.

### In vitro fertilization and embryo culture

For in vitro fertilization (IVF), high-quality semen from two bulls was purchased from a commercial artificial insemination station. The motile fraction of sperm was isolated using the BoviPure System following the manufacturer’s protocol (Nidacon, Mölndal, Sweden). Briefly, the thawed semen was centrifuged in two layers of 80% and 40% BoviPure solution at 300 *g* for 15 min, and the precipitate was subsequently washed in BoviWash solution. The final semen concentration was determined under a microscope (Nikon YS2-T) using a Burker Chamber (BRANDT), and gamete coincubation was carried out with a semen concentration of 1 × 10^6^/mL for 20 h. The in vitro fertilization medium [35] was supplemented with PHE (penicillamine, hypotaurine, epinephrine). Embryo culture was conducted in 40 μL droplets of SOF + fafBSA medium [36] for 9 days in 5% CO_2_ and 5% O_2_ and in 39°C (25–30 embryos per drop). On the third day after fertilization, half of the drop volume was replaced with fresh medium. The cleavage rate (related to the number of oocytes) was evaluated on the third day, whereas the blastocyst rate (related to the number of oocytes) and hatching rate (related to the total number of all blastocysts) were evaluated on the eighth day post-insemination.

### Lipid droplet staining, microscope analysis, and image analysis

In order to verify how FF-sEVs affect single-COC LD parameters, COCs were matured in 17 individual 20 μL droplets under oil in each replicate [31]. After maturation, COCs were washed in 0.2% PVP/PBS, and then, CCs were separated from oocytes by pipetting. Oocytes (in 4-well Nunc plates) and CCs (pooled from 17 COCs in one 1.5 mL Eppendorf tube) were fixed in 500 μL of 4% paraformaldehyde (PFA) and stored at 4°C for further analysis.

Oocytes were permeabilized in 0.2% Triton X-100 solution for 20 min at room temperature and washed in 0.2% PVP/PBS. Twenty micrograms per milliliter of BODIPY 493/503 fluorescent dye was used to stain LDs (Thermo Fisher Scientific, MA, USA). Incubation was performed in 500 μL of the dye solution in PBS at room temperature for 1 h in the dark. Oocytes were washed three times in 0.2% PVP/PBS to eject excessive dye. The chromatin of the nucleus and the polar body were visualized by staining the oocytes with 0.5 μg/mL 4′,6-diamidino-2-phenylindole (DAPI; Vector Laboratories, Burlingame, CA, USA). Oocytes were mounted on a glass slide with a single concave (Comex, Wroclaw, Poland), covered, and stored at 4°C.

Cumulus cells removed from COCs were centrifuged at 1000 *g* for 5 min, resuspended in fresh PBS, and distributed on adhesive slides (SuperFrost Menzel, ThermoFisher Scientific) using Cytospin 4 (ThermoFisher Scientific). Then, the same staining protocol as for oocytes was applied.

Oocytes and CCs were analyzed using a confocal microscope Zeiss LSM 880 using a 488 nm filter with bandpass 500–550 nm for BODIPY 493/503 (laser Argon2) and 420–480 nm for DAPI (laser Diode 405) with LD LCl Plan Apochromat 40×/1.2 Imm Korr DIC 27 objective (Zeiss, Germany). Each analyzed cell was scanned with the starting point at the top of the cell and up to the equatorial section (for oocytes) and the bottom of the cell (CCs). The intervals were 6 μm for oocytes and 2 μm for CCs. The acquired images were subjected to lipid parameter analysis, including lipid content, LD number, LD size, and the percentage of area occupied by LDs, using ImageJ Fiji software version v1.53c (NIH, Bethesda, MD, United States). The lipid content of both oocytes and CCs was determined using the formula: “integrated density” value minus (“total cell area” value × “background fluorescence” value). The cell area was determined by outlining the shape of the cell under the transmitted light for each slide separately. The measurement was then taken, and the data value was recorded in square centimeters. The remaining parameters (LD number, size, and area) were quantified through the “analyze particle” command, which identified and measured fluorescence signals above the photo background. The area occupied by LDs (%) was automatically calculated based on the fluorescence-to-non-fluorescence signal ratio. The “watershed” command was additionally employed to eliminate potential LD clusters generated by the Fiji software. For each parameter, the value of each slice was computed separately.

### Real-time qPCR analysis

Each sample of CCs and oocytes originated from a group of 25 COCs that were in vitro matured together in one well. After 22 h of IVM, COCs were transferred into PVP/PBS and denuded by vigorous pipetting. Cumulus cells were transferred into Eppendorf tubes and centrifuged (1000 *g* × 5 min), the supernatant was removed, and cells were frozen at −196°C. Fully denuded oocytes were transferred into Eppendorf tubes and frozen at −196°C. Until the beginning of experiments, samples were stored at −80°C.

Total RNA was extracted from oocytes and CC samples using the High Pure miRNA Isolation Kit (Roche, Basel, Switzerland) according to the manufacturer’s guidelines. RNA precipitation was carried out with NF Pellet Paint Co-Precipitant. For this, 1 μL of Pellet Paint, 10 μL of 3 M sodium acetate, and 200 μL of 96% ethanol were added to the RNA sample. After a 5-min incubation, the samples were centrifuged at 18 000 *g* for 10 min. The RNA pellet was washed and centrifuged in 75% and 96% ethanol and subsequently dried at 40°C. Finally, the RNA was resuspended in 8 μL of water and assessed for OD260/280 ratio values using Nanodrop 2000c (Thermo Fisher Scientific). Subsequently, reverse transcription was performed on the total isolated RNA utilizing the Transcriptor First Strand cDNA Synthesis Kit (Roche, Warsaw, Poland) following the manufacturer’s protocol. The resulting complementary DNA (cDNA) samples were diluted 1:1 in water and stored at −20°C until further analysis. The real-time qPCR analysis was conducted with the standard curve method. Initially, the product of each gene was obtained by PCR and separated on a 1.5% agarose gel. Subsequently, the PCR product was isolated and purified using the GeneJET Gel Extraction Kit (Molecular Biology, ThermoFisher Scientific). The DNA concentration was quantified using a Nanodrop 2000c (Thermo Fisher Scientific), and serial 10-fold dilutions of DNA with known concentrations (standards) were prepared. These standards were then applied in real-time qPCR reactions to generate the respective standard curves, facilitated by the LightCycler 480 II software (Roche). The reactions were run using the LightCycler 480 II system with LightCycler 480 Probes Master reagents (Roche). The 20 μL reaction mixture consisted of 10 μL LightCycler Master, 4 μL primers, 4 μL PCR-pure water, and 2 μL cDNA. The following reaction conditions were applied: denaturation at 95°C for 5 min; amplification (40 cycles) at 95°C for 10 s, 60°C for 10 s, and 72°C for 10 s; and final cooling at 40°C. Through this approach, the relative concentration of the messenger RNA (mRNA) of genes responsible for fatty acid metabolism (*FASN, PPARA, PLIN2*), glucose metabolism (*ACACA, GLUT1*)], and COC quality (*GDF9*) was evaluated (the list of primers with details—Supplementary file 1). Each cDNA sample underwent analysis in two separate PCR runs, and the resulting average value was used to determine the relative transcript abundance compared to the geometric mean of two reference genes [Glyceraldehyde 3-Phosphate Dehydrogenase (*GAPDH*)] and [Tyrosine 3-Monooxygenase/Tryptophan 5-Monooxygenase Activation Protein Zeta (*YWHAZ*)].

### Blastocyst lipid profiling

After 9 days of embryo culture, only hatched blastocysts were selected for the lipidomics experiment. A total of six single blastocysts per group, derived from three independent replicates, were included in the analysis. Each blastocyst was washed individually in PBS, transferred into an Eppendorf tube in minimum liquid volume, and frozen at −196°C. Samples were further stored at −80°C.

For lipid extraction, the collected samples were thawed and mixed with 50 μL of ultrapure water and then vortexed to induce cell lysis. Subsequently, 90 μL of methanol [high performance liquid chromatography (HPLC) grade] and 50 μL of chloroform (HPLC grade) were added and thoroughly mixed by pipetting to ensure the formation of a single-phase solution, followed by a 15-min incubation at room temperature. After incubation, 50 μL of ultrapure water and 50 μL of chloroform were added and mixed by pipetting; then, the mixture was centrifuged for 5 min at 3000 *g*. The resulting non-polar and polar phases were carefully transferred to new tubes and dried using a speedvac evaporator for approximately 4 h at room temperature. The dried lipid extract was then stored in a −80°C freezer for further analysis and experimentation.

The lipid extracts were diluted into 30 μL of acetonitrile/methanol/ammonium acetate 300 nM at the proportion of 3:6.65:0.35 (v/v/v) to reach a final concentration of 10 mM of ammonium acetate. Using a micro-autosampler (G1377A), the diluted lipid extracts were flow-injected into a triple quadrupole mass spectrometer (QQQ 6410, Agilent Technologies) at 10 μL/min using a collection of ion transitions previously reported to profile lipids present in bovine oocytes and embryos [37, 38]. The ion intensity of each ion transition (or multiple reaction monitoring) over time was obtained using in-house scripts, and, for each embryo, the list of detected lipids was generated. The maximum ion intensity of each ion transition was compared to the blank, and the lipids that did not present an ion intensity of at least 30% above the blank sample were considered noise and removed from the analysis. Obtained lipids were categorized into lipid classes, and the following analysis was performed in MetaboAnalyst 6.0 software: enrichment analysis, pathway analysis, and statistical analysis (fold change and principal component). Data were uploaded in a csv format and normalized by sum. The fold change threshold was set for 1.5.

### Extracellular vesicle PKH67 labeling

The procedure was conducted according to the protocol of PKH67 Green Fluorescent Cell linker Kit for General Cell membrane Labeling. Prior to labeling, FF-sEVs were thawed, gently vortexed, and kept on ice. To 40 μL of isolated FF-sEVs (stock concentration—98.7 mg/mL), 125 μL of DILUENT C was added and mixed by pipetting. In a separate tube, 1 μL of PKH67 dye was mixed with 250 μL of DILUENT C. The mixture was added to the FF-sEV suspension, mixed by pipetting, and incubated at room temperature for 5 min. Two negative controls were tested: (1) by adding 25 μL of PBS to 250 μL of DILUENT C with PKH67 dye and (2) by adding 25 μL of FF-sEVs to 250 μL of DILUENT C without PKH67 dye. To stop the reaction, 250 μL of EV-free FBS (Gibco, ThermoFisher Scientific) was added and incubated for 1 min at room temperature. The mixture was then transferred to tubes designated for ultracentrifugation (Thermo 5.0 mL PA Thin-Walled Tube) and centrifuged at 100 000 *g* for 30 min at 4°C (Sorvall MX120+ Micro-Ultracentrifuge). After centrifugation, the pellet of each single sample was resuspended in 20 μL of PBS.

### Extracellular vesicle uptake analysis

Labeled FF-sEVs were added to 500 μL of maturation medium in a volume of 20 μL. After 22 h of IVM, oocytes treated with FF-sEVs were incompletely denuded (leaving one to two layers of *corona radiata* cells) and fixed in 2% PFA, whereas the remaining CCs were fixed in 4% PFA. To visualize TZPs, an additional 30-min incubation in 1× phalloidin (iFluor 555 Reagent, Abcam, Cambridge, UK) was performed. Oocytes were mounted on a glass slide with a single concave (Comex, Wroclaw, Poland) with 70 μL Antifade mounting medium (ThermoFisher Scientific), covered, and analyzed the following day. Cumulus cells were mounted on the cytospin slides, centrifuged in Cytospin 4 (ThermoFisher Scientific), stained with 15 μl DAPI (Vector Laboratories, Burlingame, CA, USA), covered, and analyzed immediately.

The Zeiss LSM 880 confocal microscope was used to visualize the FF-sEV uptake. For FF-sEV labeling, the excitation wavelength was 488 nm and the emission 537 nm (laser Argon2). For DAPI staining (used only with regard to CCs), the excitation wavelength was 405 and the emission 438 nm (Laser Diode 405). For phalloidin staining, the excitation wavelength was 543 and the emission 575 nm.

### Statistical analysis

The Wilcoxon test and Kruskal–Wallis test were applied to compare statistical significance. These analyses were conducted using the statistical software package R (https://cran.r-project.org/). Comparisons between groups in the uptake experiment were performed using the chi-square test.

## Results

### Characterization of follicular-fluid small extracellular vesicles

**Figure 1:**
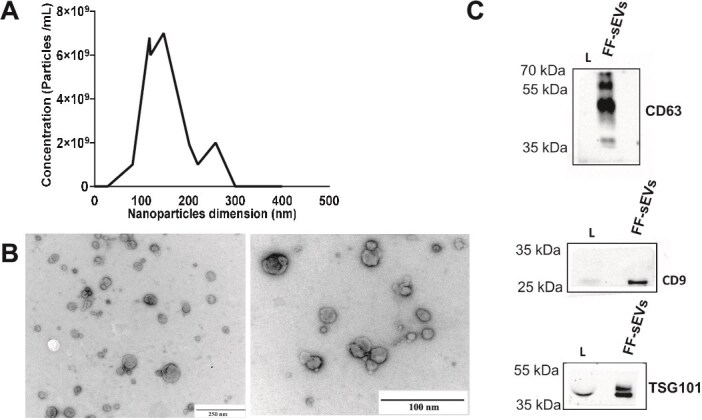
Characterization of sEVs isolated from bovine follicular fluid—concentration, morphological features, and surface markers. (A) Graph showing Nanoparticle Tracking Analysis (NTA) data of FF-sEVs concentration (particles/mL) and size distribution (nm) in pooled sample; (B) Transmission electron microscopy images of FF-sEV morphology (scale bar: 100 and 250 nm); and (C) Western blotting analysis detected the presence of extracellular vesicle-associated proteins: CD9 (25 kDa), CD63 (42 kDa), and TSG101 (42 kDa).

The NTA analysis revealed that the sizes of EV particles ranged between 30 and 450 nm (Figure 1A). The mean particle size was 166.2 ± 2 nm, with a mode of 133.5 ± 8.9 nm and a standard deviation of 68.1 ± 2.7 nm. The D50 value, representing the median particle size, was 146.6 ± 2.0 nm, while D10 and D90 values were 105.8 ± 1.5 and 257.7 ± 0.9 nm, respectively, indicating that 10% of the particles were smaller than 105.8 nm and 90% were below 257.7 nm. The concentration of nanoparticles in the sample was 1.34 × 10^10^ particles/mL. Additionally, TEM analysis revealed the presence of sEVs within a similar size range of NTA analysis (Figure 1B). Finally, WB confirmed the presence of proteins associated with EVs (CD63, CD9, and TSG101; Figure 1C).

### Enhanced follicular-fluid small extracellular vesicle uptake by cumulus cells under metabolic inhibition

**Figure 2:**
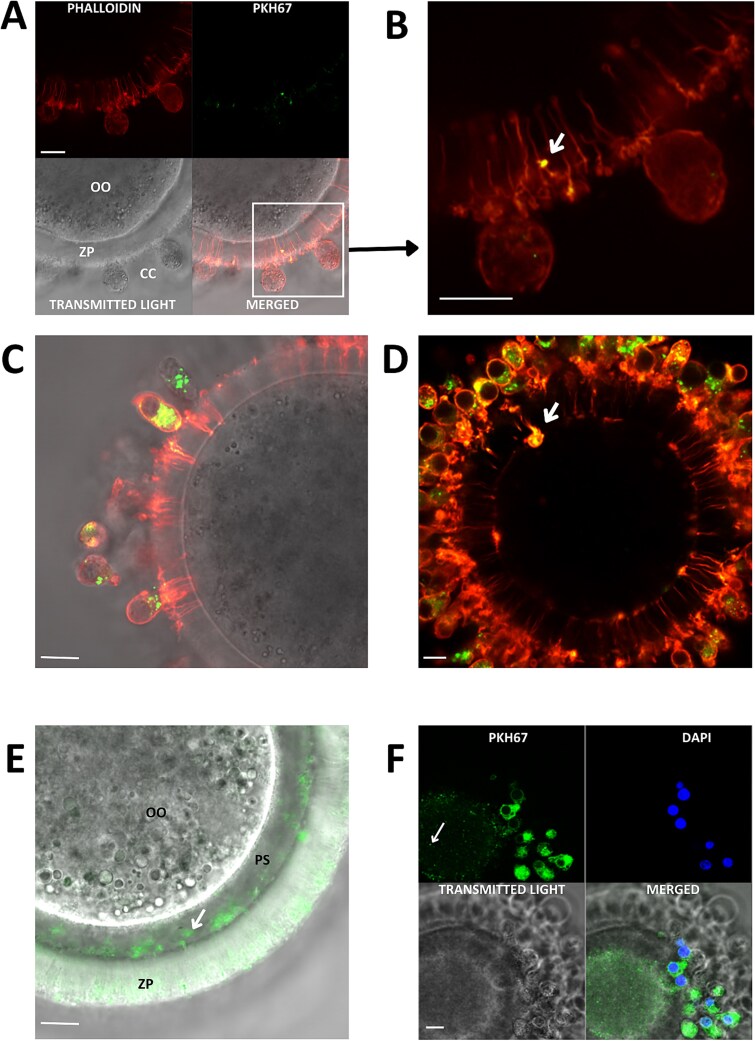
Double staining of transzonal projections (TZPs) using phalloidin (red fluorescence) and FF-sEVs labeled with PKH67 dye (green fluorescence). Signal overlap appears as a yellow color in the image. OO—oocyte, ZP—zona pellucida, CC—cumulus cells, PS—perivitelline space. Scale bar = 10 μm. All figures are representative confocal microscopy images of an oocyte with cumulus cells, with a special focus on: (A) TZPs within zona pellucida and FF-sEV signals; (B) enlarged fragment of zona pellucida with visible FF-sEVs entering the oocyte through TZPs; and (C) signals of FF-sEVs detected both in cumulus cells and within TZPs. (D) arrow indicating FF-sEVs signals next to the oocyte surface within enlarged points of TZP–oocyte attachment; (E) signals of FF-sEVs within perivitelline space indicated with an arrow; and (F) signals of FF-sEVs in the cytoplasm of the oocyte indicated with an arrow.

**Figure 3:**
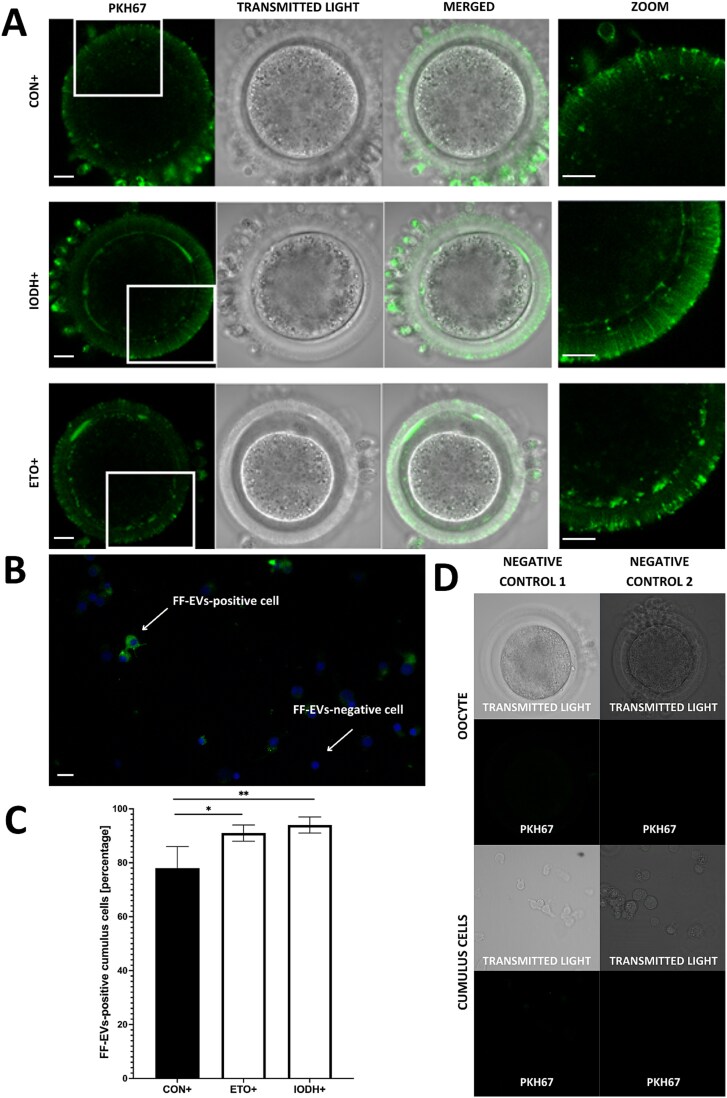
The effect of FF-sEV uptake after 22 h of COC maturation. Experimental groups: CON+ (control), IODH+ (maturation with glucose metabolism inhibitors), and ETO+ (maturation with a lipid metabolism inhibitor). Green fluorescence represents PKH67-labeled FF-sEVs, while blue fluorescence indicates DAPI-stained nuclei. Scale bar = 10 μm. (A) Confocal images of oocytes of the three supplemented groups. (B) Confocal images of cumulus cells with or without internalized FF-sEVs. (C) Bar graph showing the percentage of FF-sEV-positive cumulus cells in the three analyzed groups (mean ± SD; ^*^P ≤ 0.05, ^**^P ≤ 0.01); (D) Confocal images of oocytes and cumulus cells from two negative controls: (1) labeling procedure without FF-sEVs, (2) labeling procedure without PKH67 dye.

Two approaches were used to analyze the FF-sEV uptake in bovine COCs. For the analysis of FF-sEV uptake within cumulus–oocyte complexes, 10–15 partially denuded COCs from each group (CON+, IODH+, ETO+) were scanned in three replicates of each. Phalloidin staining allowed us to visualize FF-sEVs, which crossed the zona pellucida through TZPs (Figure 2). Follicular-fluid extracellular vesicle signals were also detected next to the oocyte surface within enlarged points of TZP–oocyte attachment (Figure 2D). The vesicles were further present in the perivitelline space (Figure 2E), and ooplasm (Figure 2F). Moreover, confocal microscopy images of oocytes revealed the presence of FF-sEVs both in oocytes and CCs in control as well as two experimental groups (Figure 3A).

For quantitative measurements of FF-sEV uptake in CCs, a total of 3797 cells were analyzed across three groups, with three replicates in each group: CON+ (*n* = 1188), ETO+ (*n* = 1314), and IODH+ (*n* = 1295). The analysis focused on the ratio of EV-positive CCs (cells that accumulated FF-sEVs within the cytoplasm) in relation to the total number of cells evaluated (FF-sEV-positive and negative cells visualized in Figure 3B). Both in IODH+ (94% ± 3, *P* ≤ 0.01) and ETO+ (92% ± 3, *P* ≤ 0.05), significantly more CCs accumulated FF-sEVs when compared to the control (79% ± 7, Figure 3C).

### Follicular-fluid small extracellular vesicles enhance oocyte maturation and blastocyst hatching in inhibited metabolism conditions

**Figure 4:**
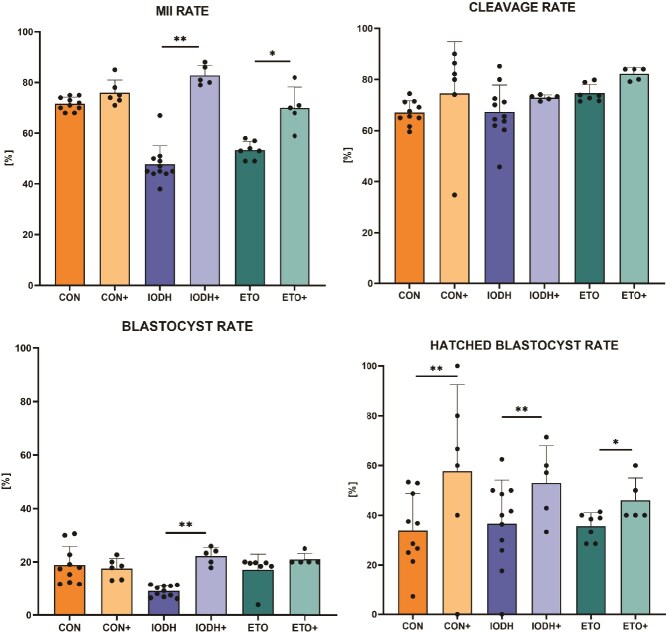
The effect of FF-sEV supplementation on the in vitro production outcome. The metaphase II (MII), cleavage, blastocyst, and hatched blastocyst rates [%], compared between supplemented and non-supplemented groups (means ± SD). Metaphase II, cleavage, and blastocyst rates are related to the number of immature oocytes, whereas the hatched blastocyst rate is related to the number of all blastocysts. ^*^P ≤ 0.05, ^**^P ≤ 0.01.

The in vitro production experiments were based on a specific number of experimental replicates per group (CON – 10, CON+ − 6, IODH – 11, IODH+ − 5, ETO – 7, ETO+ − 5). Follicular-fluid sEV supplementation had no significant impact on metaphase II (MII) rate in the control conditions (76% and 72%, CON+ vs CON, respectively). However, supplementation of maturation media with FF-sEVs along with lipid or glucose metabolism inhibitors significantly increased the MII rate in both experimental groups: from 48% to 83% in IODH+ (*P* < 0.01) and from 53% to 70% in ETO+ (*P* < 0.05) when compared to their controls (IODH and ETO, respectively) (Figure 4).

There were no significant differences in the cleavage rate across treatment groups. Regarding the blastocyst rate calculated relative to the total number of immature oocytes, only the IODH+ group showed a statistically significant increase (*P* < 0.01) when compared to the non-supplemented group (22% and 9% blastocyst rate, respectively). Significant differences were observed in the hatched blastocyst rates among all supplemented vs non-supplemented groups. The CON+, IODH+, and ETO+ groups showed increases of 24% (*P* < 0.01), 17% (*P* < 0.01) and 10% (*P* < 0.05) (Figure 4).

### Follicular-fluid small extracellular vesicles modulate lipid droplets in cumulus cells and oocytes

**Figure 5:**
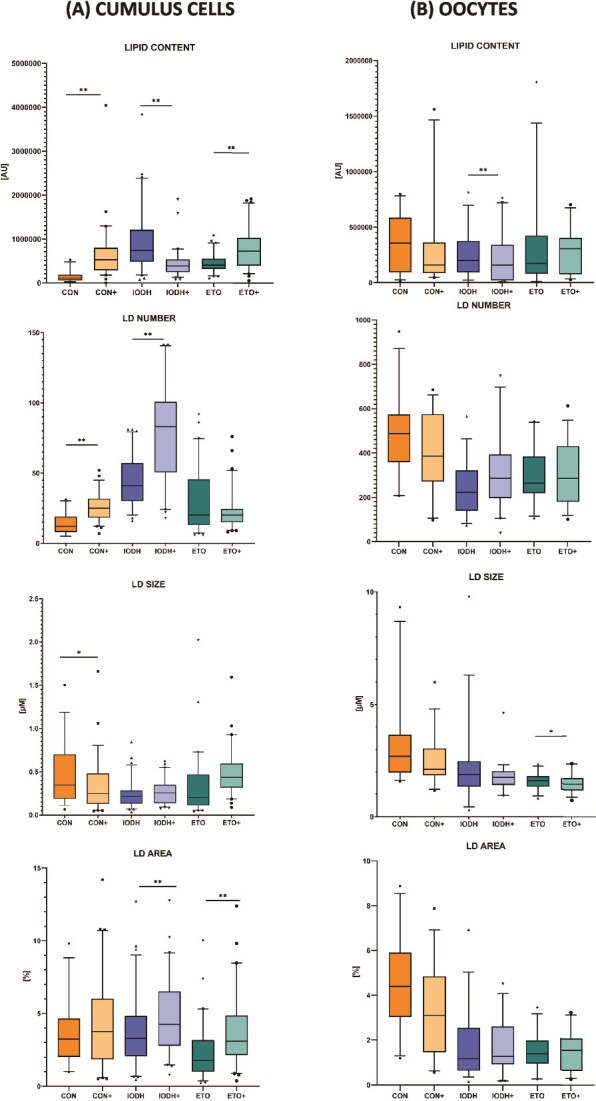
Lipid droplet parameters in oocytes and cumulus cells. The comparison is revealed between supplemented and non-supplemented groups (CON+ vs CON, IODH+ vs IODH, ETO+ vs ETO) for cumulus cells (A, diagrams in left column) and oocytes (B, diagrams in right column); ^*^P ≤ 0.05, ^**^P ≤ 0.01.

Lipid droplets were analyzed in 366 CCs (CON *n* = 60; CON+ *n* = 60; IODH *n* = 63; IODH+ *n* = 60; ETO *n* = 61; ETO+ *n* = 62) and 243 oocytes (CON *n* = 38; CON+ *n* = 39; IODH *n* = 48; IODH+ *n* = 40; ETO *n* = 38; ETO+ *n* = 40) in four replicates. Four LD parameters were assessed: lipid content, LD size, number of LDs, and LD area (Figure 5).

In CCs, comparing the CON+ with the CON group, the supplementation of FF-sEVs increased lipid content and LD number and also decreased LD size. In the IODH group, FF-sEV supplementation led to a decrease in lipid content and an increase in LD number and LD area, with no alterations in LD size. In the ETO group, FF-sEV supplementation increased lipid content and LD area, with no changes in LD number or size.

In oocytes, no differences were detected between the CON+ and CON groups across any of the analyzed parameters. Comparing IODH+ versus IODH conditions, observations revealed a decrease in lipid content after FF-sEV supplementation. Similarly, in the comparison between ETO+ and ETO, LD size showed a notable decrease upon supplementation.

### Follicular-fluid small extracellular vesicle supplementation affects relative transcript levels of energy metabolism–related genes both in oocytes and cumulus cells

**Figure 6:**
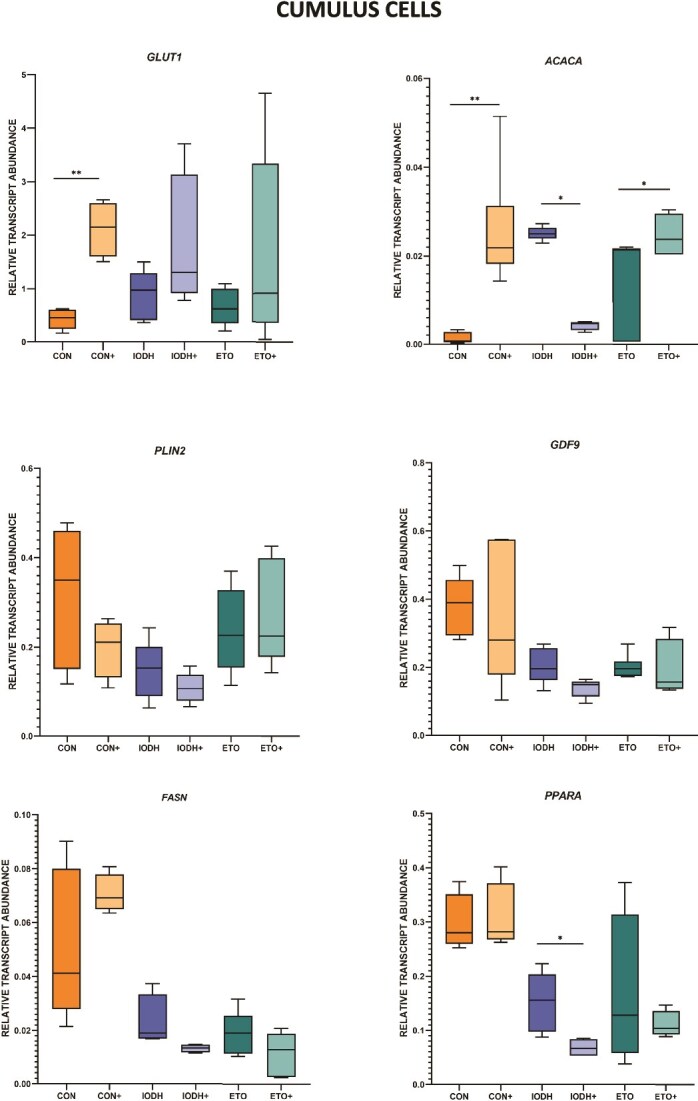
The effect of FF-sEV supplementation on the relative mRNA level of selected genes in cumulus cells. The comparison is revealed between supplemented compared to non-supplemented groups (CON+ vs CON, IODH+ vs IODH, ETO+ vs ETO). The results are shown as a transcript abundance of the gene of interest related to the geometric mean of reference genes. ^*^P ≤ 0.05, ^**^P ≤ 0.01.

**Figure 7:**
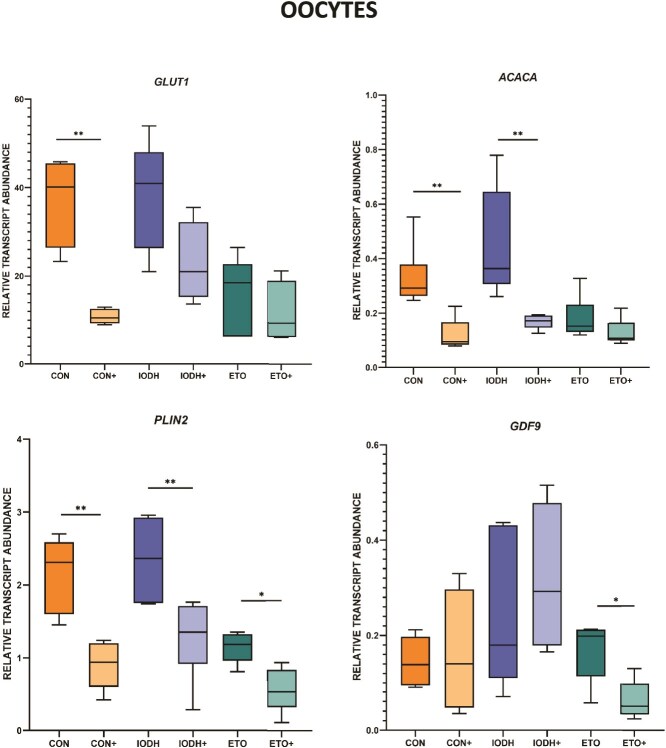
The effect of FF-sEV supplementation on the relative mRNA level of selected genes in oocytes. The comparison is revealed between supplemented compared to non-supplemented groups (CON+ vs CON, IODH+ vs IODH, ETO+ vs ETO). The results are shown as a transcript abundance of the gene of interest related to the geometric mean of reference genes. ^*^P ≤ 0.05, ^**^P ≤ 0.01.

Relative transcript levels for selected genes involved in energy metabolism were analyzed in 36 samples of CCs (six replicates per group; Figure 6) and 36 samples of oocytes (six replicates per group; Figure 7).

In CCs, CON+ showed significant upregulation of *GLUT1* and *ACACA* (*P* < 0.01) mRNA levels. During maturation with glucose metabolism inhibitors, supplementation with FF-sEVs resulted in downregulation of *ACACA* (*P* < 0.05) and *PPARA* (*P* < 0.05) mRNA levels. When fatty acid metabolism inhibition was applied, the sole significant observation was an upregulation of *ACACA* in ETO+ vs ETO (*P* < 0.05).

In the oocytes, the CON+ group presented decreased mRNA levels of *GLUT1*, *ACACA,* and *PLIN2* (*P* < 0.01), with *GDF9* remaining unaffected. A decrease of *ACACA* and PLIN2 mRNA levels in IODH+ vs IODH was observed. In ETO+ vs ETO, a significant decrease in *GDF9* expression was observed. The absence of *FASN* and *PPARA* in this analysis is due to their mRNA concentrations being below the threshold of reliable detection, falling within a range that approaches the limit of assay sensitivity.

### Follicular-fluid small extracellular vesicle supplementation during in vitro maturation alters lipid profiles and metabolic pathways in blastocysts

The lipidomic analyses were conducted on blastocysts originating from oocytes matured in the experimental groups—both supplemented with FF-sEVs (IODH+, *n* = 6; ETO+, *n* = 6) and non-supplemented (IODH, *n* = 6; ETO, *n* = 6). The analysis resulted in the identification of a total of 207 distinct lipid species classified in nine subclasses (the number of lipid species within each lipid subclass is shown in the brackets): PC—phosphatidylcholine (52), PE—phosphatidylethanolamine (48), TGs—triglycerides (42), acyl-carnitines (19), PS—phosphatidylserine (15), PG—phosphatidylglycerol (11), SM—sphingomyelin (7), PI—phosphatidylinositol (7), CE—cholesterol esters (6). Three of the most abundant lipid classes in the analyzed blastocysts were PC, PE, and triacylglycerols.

Using MetaboAnalyst 6.0 software, IODH+ vs IODH and ETO+ vs ETO were compared. Specifically, fold change analysis, enrichment analysis, and principal component analysis (PCA) were performed to detect differential lipid profiles and potential metabolic pathways affected in these experimental conditions.

**Figure 8:**
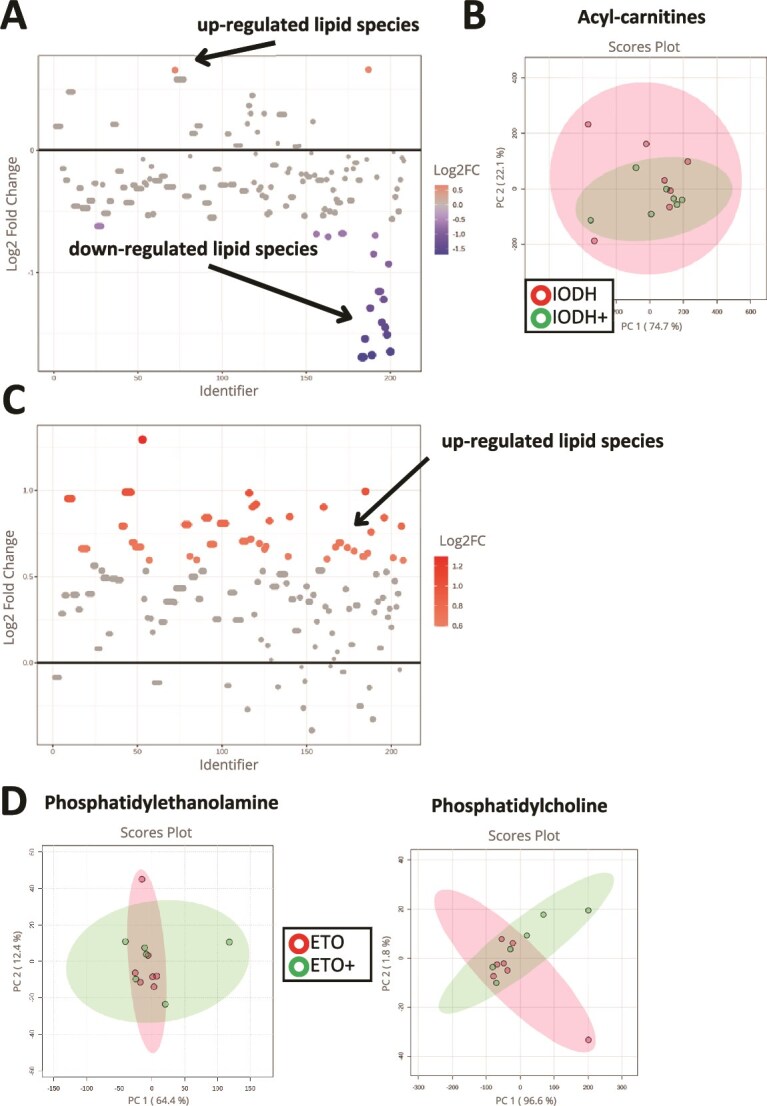
Lipidomics response of the blastocysts on FF-sEV supplementation during IVM originated from oocytes matured in medium supplemented with inhibitors of glucose metabolism (A and B) or inhibitors of lipid metabolism (B and C). (A, C) Fold change analysis graphs of detected lipids; (B, D) Score plot analysis of lipid groups that were the most numerous groups of lipids significantly highlighted in the fold change analysis.

To evaluate the impact of FF-sEV supplementation on the blastocyst lipidome in IODH+ vs IODH groups, statistical and enrichment analyses were conducted. Specifically, 22 lipid species were found to be downregulated, while 2 lipid species were upregulated in the IODH+ group when compared to the IODH group (Supplementary file 2). Among the downregulated lipid species, the most abundant subclass was acyl-carnitines, with 15 members exhibiting decreased concentration levels. Additionally, four TGs, three PCs, and one PE were also found to be significantly changed (Figure 8A). Upregulated lipids belonged to PEs (1) and acyl-carnitines (1). The PCA was conducted specifically on the acyl-carnitines group, which exhibited the most significant changes. The results revealed 74.6% of the diversity within this subclass. The enrichment analysis pointed out that the two pathways’ activities significantly changed: phospholipid biosynthesis and arachidonic acid metabolism.

In the comparison of ETO+ and ETO conditions on blastocyst lipidome (Figure 8C), a total of 65 lipid species (Supplementary file 3) were found to be significantly upregulated. Among these upregulated lipid species, PCs were the most prominent group, with 19 members showing increased content. Additionally, PEs exhibited significant upregulation, with 13 members showing increased content. The PCA was conducted separately for the PE and PC lipid subclasses. The outcome showed 64.4% and 96.6% of the diversity within these classes, respectively. The pathway analysis identified significant enrichment of phospholipid biosynthesis and arachidonic acid metabolism, in conformity with the results observed for IODH+.

## Discussion

The fundamental element of the present experiment was a proper isolation of FF-derived small EVs and verification of their quality as well as quantity. The characterization of FF-sEVs isolated for this research was performed according to guidelines proposed in Minimal information for studies of extracellular vesicles (MISEV) 2023 [39]. The NTA and TEM analyses identified FF-sEVs with a mean size of 166.2 +/− 2.0 nm and a mean concentration of 1.34 × 10^10^ particles/mL. These findings are consistent with previous studies in bovine [14, 40], humans [41], buffalo [42], and equine [43].

Confocal imaging shows that FF-sEVs are initially incorporated by CCs and subsequently transferred to the oocyte. Our findings are in accordance with a previous study in cattle, in which fluorescently labeled EVs were noticed in CCs and inside the zona pellucida [11]. However, in the current study, we demonstrate for the first time that FF-sEVs are present within TZPs. For TZP visualization, staining of phalloidin was applied, which is a cyclic heptapeptide that binds to filamentous (F)-actin, an element of TZPs [44]. On the images obtained from the confocal microscopy, we demonstrate signals of FF-sEVs detected within TZPs passing through the zona pellucida (Figure 2A and B). We also detect FF-sEV signals next to the oocyte surface within enlarged points of TZP–oocyte attachment (Figure 2C), which were described as a common extension of TZPs toward the oocyte [44]. Further, we also detect FF-sEV signals in the perivitelline space (Figure 2D) and in the oocyte cytoplasm (Figure 2E). PKH67, which is a dye used in the present experiment for FF-sEV labeling, is a highly fluorescent, lipophilic, long-chain carbocyanine dye used to stain EV membranes [45]. Obtained images indicate that FF-sEV structures detected and visualized in the present study are intact and are transported from maturation medium to the oocyte via TZPs.

Further, we demonstrated that CCs within COCs exhibit significantly higher uptake of FF-sEVs under induced metabolic stress when compared to the control group. This increased uptake is observed both under conditions of glucose metabolism inhibition and fatty acid metabolism inhibition. It has been previously described that cells release EVs in response to stress stimuli in order to spread signaling molecules or to remove waste material [46]. Furthermore, the content of EVs varies in response to stress factors (e.g., oxidative stress), subsequently influencing the recipient cells’ ability to withstand unfavorable conditions and maintain viability [47]. To the best of our knowledge, we demonstrate for the first time an increase in FF-sEV uptake by CCs as a response to metabolic stress conditions during IVM, suggesting that FF-sEVs may support the counteraction of the negative effects of the environment.

The CC layer acts as a molecular filter, selecting specific types and amounts of small metabolites to be transported to the oocyte [48], which may have a beneficial effect. Follicular-fluid EVs are probably involved in this mechanism, as they have been shown to influence the viability, expansion, and gene expression (transcriptome) of equine CCs [49]. This hypothesis is supported by the present study, where FF-sEV supplementation during metabolic stress conditions significantly changes relative transcript levels of lipid metabolism–related genes along with LD parameters in CCs. Changes in gene expression and lipid composition in response to metabolic stress after FF-sEVs supplementation were more pronounced in CCs than in oocytes. The most significant alterations occurred when glucose metabolism was inhibited, since FF-sEV supplementation reduced the transcript levels of *ACACA* and *PPARA* genes in CCs. Additionally, a decreased total lipid content was observed, suggesting that FF-sEVs supplementation may enhance the utilization of LDs for energy production to compensate for the reduced glycolysis. The decreased *ACACA* transcript level may be a consequence of this mechanism, as this gene encodes a key enzyme involved in the biosynthesis of long-chain fatty acids [50], a process typically activated mainly when excess energy is available. Furthermore, the downregulation of the *PPARA* gene in CCs following FF-sEV supplementation suggests a reduction in fatty acid uptake, which may also limit the accumulation of external energy reserves in LDs. In our opinion obtained results suggest that FF-EV supplementation enhances the production of energy from lipid sources under glucose metabolism disturbances in CCs. In contrast to CCs, there were almost no changes observed in LD parameters in oocytes when supplemented vs non-supplemented groups were compared. Surprisingly, with regard to gene expression results, most of the analyzed genes were down-regulated in at least one of the FF-sEV-supplemented groups; however, no clear pattern of response was observed. In our opinion, the observed outcomes highlight the complexity of the process involved, suggesting that further studies are needed to achieve a more comprehensive understanding of the EVs’ role in COC metabolism.

Analyzing the obtained results in the context of oocyte quality, it is clearly demonstrated that supplementation with FF-sEVs during IVM has a significant impact on maturing oocytes. Under metabolic stress, FF-EV supplementation resulted in significantly higher MII rates. This is in conformity with previous studies where FF-EV supplementation supported equine oocyte maturation [43] and enhanced meiotic resumption after vitrification of feline COCs [17]. Proteomic analysis of feline FF-sEVs identifies 674 proteins involved in the regulation of many pathways, including oxidative phosphorylation, extracellular matrix formation, oocyte meiosis, cholesterol metabolism, glycolysis/gluconeogenesis, MAPK, PI3K-AKT, HIPPO, and calcium signaling pathways [17]. It may be therefore suggested that an increased uptake of FF-sEVs in response to metabolic stress in the present study could support better meiosis progression and improved COC quality, acting within these pathways.

We recently demonstrated that supplementation of IVM medium with selected microRNA identified in FF-EVs significantly improved blastocyst quality with a higher total cell number and inner cell mass number [51]. In the current study, FF-sEV supplementation during IVM improved both development rates as well as embryo quality. The blastocyst rate was significantly improved in the IODH+ group, whereas the hatching rate was enhanced across all supplemented groups vs their non-supplemented controls (Figure 3). Our previous experiments have already revealed that the oocyte maturation environment not only affects the oocytes and CCs’ metabolism [23] but also exerts a substantial effect on preimplantation development programming at cellular and molecular levels [52]. We showed that inhibition of glucose and fatty acid metabolism led to cellular stress response, compromising the quality of preimplantation embryos [52]. Since previously published results showed limited embryo development when glucose metabolism was inhibited during the IVM stage [22], the present results indicate that FF-sEV supplementation not only supports the maturation process but also enhances oocyte and embryo developmental competence. Changes observed in blastocysts in the present study, including the hatching rate, are a biologically plausible outcome consistent with findings reported in other studies [32]. These conclusions are further supported by the results of our lipidomic study.

The lipidome pathway enrichment analysis revealed that the same two metabolic pathways are up-regulated by FF-sEVs supplementation in both inhibition systems: phospholipid biosynthesis and arachidonic acid metabolism. These pathways are integral to the structure and functionality of cellular membranes [53–55]. Furthermore, they are involved in various biological processes, such as cellular defense, repair, and inflammation (through arachidonic acid) [56]. Consequently, it can be inferred that FF-sEVs uptaken during IVM support the proliferation of further blastocysts by promoting cell division, which aligns with our previous study showing lower blastomere numbers within the trophectoderm in IODH and ETO blastocysts [52]. By modulating the arachidonic acid metabolism pathway, FF-sEVs may enhance implantation during further embryo development. This hypothesis is supported by the increased hatching rate observed in all FF-sEV-supplemented groups.

Lipidomic analysis under metabolic stress identified acyl-carnitines as the most abundant class of lipids, with significantly decreased concentrations of these lipid species in IODH+ blastocysts. Acyl-carnitines play an essential regulatory role in the balance between intracellular sugar and lipid metabolism by serving as carriers to transport activated long-chain fatty acids into mitochondria for β-oxidation [57]. Therefore, downregulation of acyl-carnitines within the blastocyst cells may affect overall metabolic homeostasis toward excessive accumulation of lipids.

In response to lipid metabolism inhibition, two lipid subclasses were identified as being the most significantly upregulated in ETO+ blastocysts: PC and PE. These phospholipids are among the most abundant components of mammalian cell membranes, sharing critical roles as structural elements, participants in lipid metabolism and signaling pathways, contributors to membrane trafficking and autophagy, and mediators of protein interactions [58, 59]. The increased concentration of these lipids likely supports the maintenance of a balanced lipid composition in cell membranes, which is essential for optimal membrane dynamics, signaling, and metabolic functions. Furthermore, the elevated levels of PC and PE may enhance the cell’s ability to manage stress conditions through improved autophagy, potentially leading to increased cell survival and functionality under adverse conditions. These outcomes indicate that FF-EV supplementation during IVM results in blastocysts with a greater ability to withstand metabolic stress and may promote cell divisions. This is further supported by a higher hatching rate in ETO+ blastocysts in this study.

## Conclusions

Our study provides novel insights into the role of FF-sEVs in oocyte maturation and blastocyst development. We demonstrate that FF-sEVs are transported from the culture medium into CCs and through TZPs into the perivitelline space and ooplasm, revealing EV-mediated communication within the maturation environment. We show for the first time that CCs exhibit an increased uptake of FF-sEVs from the maturation medium under metabolic stress conditions. Furthermore, FF-sEV supplementation during stress conditions enhances the MII rate in oocytes and positively affects subsequent embryo quality, as evidenced by improved hatching rates and favorable lipidomic alterations in blastocysts. Lipid droplet parameters and gene expression in both CCs and oocytes matured under metabolic stress conditions are affected by FF-sEV supplementation. These effects are more pronounced in CCs, suggesting that FF-sEVs play a supportive role in determining oocyte quality by acting through CCs. Overall, FF-sEV supplementation during IVM under metabolic stress conditions has a positive impact on the COCs and blastocysts. In the future, obtained results may help to optimize the maturation conditions of oocytes collected from females burdened with severe metabolic disorders like obesity or diabetes.

## Supplementary Material

Supplementary_file_1_ioaf096

Supplementary_file_2_ioaf096

Supplementary_file_3_ioaf096

## Data Availability

The data underlying this article will be shared on reasonable request to the corresponding author.
