# Supplementary material for: Follicular-fluid extracellular vesicles support energy metabolism of bovine oocytes, improving blastocyst development and quality
Source: Biol Reprod. 2025 Apr 24;113(1):109–26. doi: 10.1093/biolre/ioaf096 (PMC12260498; doi:10.1093/biolre/ioaf096)
Supplement: Supplementary_file_1_ioaf096 [file supplementary_file_1_ioaf096.pdf]

Supplementary file 1. The sequences of primers, length, and efficiency of products obtained from mRNA gene expression experiment. The genes listed below were analyzed in oocytes and cumulus cells. F – forward primer sequence, R – reverse primer sequence.

Gene	Sequences of Primers (F, R)	Length (bp)	Efficiency
FASN	F – CTGCCGAAGACAGGGATTG<br>R – CTGGTATACCTTCCCGCTCG	**108**	**1.822**
PPARA	F – AGTGCCTTTCAGTTGGGATGTC<br>R – CGCGGTTTCGGAATCTTCTA	**125**	**2.179**
ACACA	F – GGGAACCGTGAAGGCCTA<br>R – TGGACCAAGCTGCGGAT	**159**	**2.052**
PLIN2	F – GAGTGGAAGAGAAGCATCGG<br>R – GTGACTCAATGTGCTCAGCA	**67**	**1.91**
GDF9	F – AGCGCCCTCACTGCTTCTATAT<br>R – TTCCTTTTAGGGTGGAGGGAA	**80**	**2.079**
Glut1	F – ATCCTCATTGCCGTGGTGCT<br>R – ACGATGCCAGAGCCGATGGT	**133**	**1.964**
GAPDH	F – ACCCTCAAGATTGTCAGCAA<br>R – GCGTGGACAGTGGTCATAAG	**113**	**2.035**

YWHAZ	F – TGAACTCCCCTGAGAAAGCCT	**149**	**2.079**
	R – ATCCGATGTCCACAATGTCAAG		
