# Supplementary material for: Follicular-fluid extracellular vesicles support energy metabolism of bovine oocytes, improving blastocyst development and quality
Source: Biol Reprod. 2025 Apr 24;113(1):109–26. doi: 10.1093/biolre/ioaf096 (PMC12260498; doi:10.1093/biolre/ioaf096)
Supplement: Supplementary_file_2_ioaf096 [file supplementary_file_2_ioaf096.pdf]

Column1	Column2
**Lipid species**	**Fold Change**
CAR(22:0)	1.5815
PE(38:6)	1.5765
PC(34:2)	0.65036
PC(O-35:2)	0.65036
PC(P-35:1)	0.65036
TG(52:0)	0.62366
TG(53:7)	0.62366
TG(55:11)	0.6218
(9Z)-3-hydroxyoctadecenoylcarnitine	0.61664
TG(55:8)	0.61264
CAR(18:1)	0.55492
CAR(18:4)	0.52432
12-Hydroxy-12-octadecanoylcarnitine	0.44878
3-hydroxyoctadecanoylcarnitine	0.44878
O-(13-carboxytridecanoyl)carnitine	0.42861
CAR(20:0)	0.40841
O-(11-carboxyundecanoyl)carnitine	0.37613
CAR(17:0)	0.36545
3-hydroxyarachidonoylcarnitine	0.35104
CAR(16:0)	0.34281
CAR(22:4)	0.31879
O-(17-carboxyheptadecanoyl)carnitine	0.31263
2-Hydroxymyristoylcarnitine	0.3089
3-hydroxytetradecanoylcarnitine	0.3089
