# Supplementary material for: Follicular-fluid extracellular vesicles support energy metabolism of bovine oocytes, improving blastocyst development and quality
Source: Biol Reprod. 2025 Apr 24;113(1):109–26. doi: 10.1093/biolre/ioaf096 (PMC12260498; doi:10.1093/biolre/ioaf096)
Supplement: Supplementary_file_3_ioaf096 [file supplementary_file_3_ioaf096.pdf]

Column1	Column2
Lipid species	Fold Change
SM(d16:1/18:0)	2.4516
CAR(16:0)	1.9904
PC(36:1)	1.9844
PC(O-37:1)	1.9844
PC(O-38:8)	1.9844
PC(P-37:0)	1.9844
PS(O-41:0)	1.978
PC(34:1)	1.934
PC(O-35:1)	1.934
PC(P-35:0)	1.934
PS(O-18:0)	1.8914
PS(P-36:0)	1.8697
TG(52:1)	1.8686
PG(P-35:1)	1.7975
O-(13-carboxytridecanoyl)carnitine	1.7927
PE(33:2)	1.7905
PE(O-34:2)	1.7905
PE(P-34:1)	1.7905
PS(O-16:0)	1.7678
PE(36:1)	1.7496
PE(O-37:1)	1.7496
PE(O-38:8)	1.7496
PE(P-37:0)	1.7496
PE(35:5)	1.7416
PE(O-36:5)	1.7416
PE(P-36:4)	1.7416
CE(18:1)	1.7317
PC(34:3)	1.7316
PC(P-35:2)	1.7316
CAR(20:0)	1.6908
PS(P-33:1)	1.6435
PI(39:4)	1.6295
PI(O-40:4)	1.6295
PI(P-40:3)	1.6295
PC(30:0)	1.6221
PC(O-31:0)	1.6221
TG(48:1)	1.6202
TG(49:8)	1.6202
PS(O-38:0)	1.6147
PE(38:2)	1.6091
PE(O-40:9)	1.6091
PE(P-39:1)	1.6091
PS(P-31:1)	1.5968
PC(35:1)	1.5923
PC(36:8)	1.5923
PC(O-36:1)	1.5923

PC(P-36:0)	1.5923
TG(51:9)	1.5918
TG(53:10)	1.5883
PC(36:2)	1.5814
PC(O-37:2)	1.5814
PC(O-38:9)	1.5814
PC(P-37:1)	1.5814
PS(P-29:0)	1.5783
TG(48:2)	1.5661
CAR(8:0)	1.5532
PE(38:5)	1.5339
3-hydroxytetradecanoylcarnitine	1.5332
2-Hydroxymyristoylcarnitine	1.5332
PG(O-35:2)	1.5328
(9Z.12Z.15Z)-3-hydroxyoctadecatrienoylcarnitine	1.5246
TG(54:1)	1.5172
PE(33:1)	1.5121
SM(d18:1/24:1(15Z))	1.5105
CE(22:5)	1.5092
